# Remdesivir use and antimicrobial stewardship restrictions during the coronavirus disease 2019 (COVID-19) pandemic in the United States: A cross-sectional survey

**DOI:** 10.1017/ash.2023.146

**Published:** 2023-03-31

**Authors:** Alfredo J. Mena Lora, Rodrigo Burgos, Scott Borgetti, Lelia H. Chaisson, Susan C. Bleasdale

**Affiliations:** University of Illinois at Chicago, Chicago, Illinois

## Abstract

Deploying therapeutics for coronavirus disease 2019 (COVID-19) has proved challenging due to evolving evidence, supply shortages, and conflicting guideline recommendations. We conducted a survey on remdesivir use and the role of stewardship. Use differs significantly from guidelines. Hospitals with remdesivir restrictions were more guideline concordant. Formulary restrictions can be important for pandemic response.

Remdesivir (RDV) was the first agent with proven clinical benefit against coronavirus disease 2019 (COVID-19).^1,2^ Despite widespread use in the United States, RDV remains controversial. Some studies have shown that RDV can shorten hospitalization days for patients with admitted with hypoxia due to COVID-19, but others have found that it does not improve outcomes.^1–4^ RDV has a proven benefit in patients requiring low-flow nasal canula (LFNC), however, no benefit has been found for mechanically ventilated patients, and data for those requiring high-flow nasal canula (HFNC) and noninvasive positive pressure ventilation (NIPPV) are conflicting.^1–5^ Multiple studies, including the SOLIDARITY and DisCoVeRy trials, have reported conflicting results on who benefits from RDV.^5,6^

Deploying novel therapeutics during the COVID-19 pandemic has proven challenging, with evolving literature, supply shortages, and conflicting guidelines.^2–4^ Antimicrobial stewardship programs (ASPs) can help guide antimicrobial use, improve patient outcomes, and reduce costs. Thus, the ASP is uniquely positioned to provide guidance on appropriate implementation of novel therapeutics. In this study, we evaluated RDV utilization practices throughout the United States during the COVID-19 pandemic, alignment with evidence-based guidelines, and the role of ASPs in RDV allocation in US hospitals.

## Methods

### Study design and participants

We conducted a cross-sectional survey to assess RDV utilization practices and the role of ASP at US hospitals. We developed a web-based survey in REDCap to capture data on characteristics of survey respondents (eg, respondent’s job type), hospitals represented (eg, hospital size and type), and hospital therapeutic utilization practices throughout four waves of the COVID-19 pandemic, including RDV use and formulary restrictions implemented via ASP (Supplement 1). We requested data on RDV use over 4 COVID-19 waves: the first wave (April–June 2020), first winter wave (November 2020–February 2021), the SARS-CoV-2 δ (delta) wave (August–November 2021) and the SARS-CoV-2 ο (omicron) wave (December 2021–February 2022) (Supplement 2). The University of Illinois at Chicago Institutional Review Board approved this study.

On April 22 and 25, 2022, we recruited participants by distributing the survey link via online messaging boards and listservs for members of the Infectious Diseases Society of America (IDSA) IDea network, the IDSA Antimicrobial Stewardship Centers for Excellence, and the Society for Healthcare Epidemiology. The survey remained open through May 15, 2022. Participants submitted responses anonymously.

### Data analysis

For this analysis, we included survey data from respondents based at US hospitals, and we excluded surveys that did not report on RDV use across all 4 COVID-19 waves. To explore RDV use, we used descriptive statistics to assess (1) characteristics of survey respondents and hospitals represented; (2) RDV restrictions and characteristics of hospitals with and without RDV restrictions; and (3) RDV use in patients of varying disease severity, based on oxygen needs. We stratified results by pandemic wave. We used National Institutes of Health guidelines to define evidence-based use.^2^ Throughout the pandemic, these guidelines recommend RDV for patients requiring oxygen via LFNC and recommend against RDV in patients requiring mechanical ventilation (MV). In patients requiring HFNC or NIPPV, RDV use is controversial. The guidelines note that clinical trial data have not established a clear benefit of RDV in this group, but they postulate that it may have a role in some patients, such as immunocompromised hosts. Thus, the routine use of RDV on all patients requiring HFNC and NIPPV is not advised due to lack of robust evidence.

## Results

### Survey respondents

In total, 99 survey responses were received, of which 21 were excluded due to incomplete data: 5 from international sites and 16. Of the 78 surveys included from respondents at 78 unique hospitals, and 28 states and 44 cities were represented (Supplementary Material 3). Survey respondents included 50 infectious diseases (ID) physicians (64%), 19 pharmacists (24%), 2 hospitalists (3%), and 7 infection preventionists (9%). Also, 13 hospitals (16%) had <200 beds, 14 (18%) had 201–300 beds, 12 (15%) had 301–400 beds, and 39 (50%) had >400 beds (Table 1).

**Table 1.** Hospital Characteristics and Remdesivir Therapeutic Restrictions

Characteristic	RDV restrictions (N=41), No. (%)	No RDV restrictions (N=37), No. (%)
**Hospital type**		
Community	10 (25)	19 (51)
County	2 (5)	1 (3)
University	19 (46)	12 (33)
University affiliated	5 (12)	2 (5)
Other	5 (12)	3 (8)
**Hospital size**		
<200 beds	7 (17)	6 (16)
201–300 beds	8 (19)	6 (16)
301–400 beds	6 (15)	6 (16)
>400 beds	20 (49)	19 (52)

Note. RDV, remdesivir.

### Remdesivir use and antimicrobial stewardship programs

**Fig. 1.**
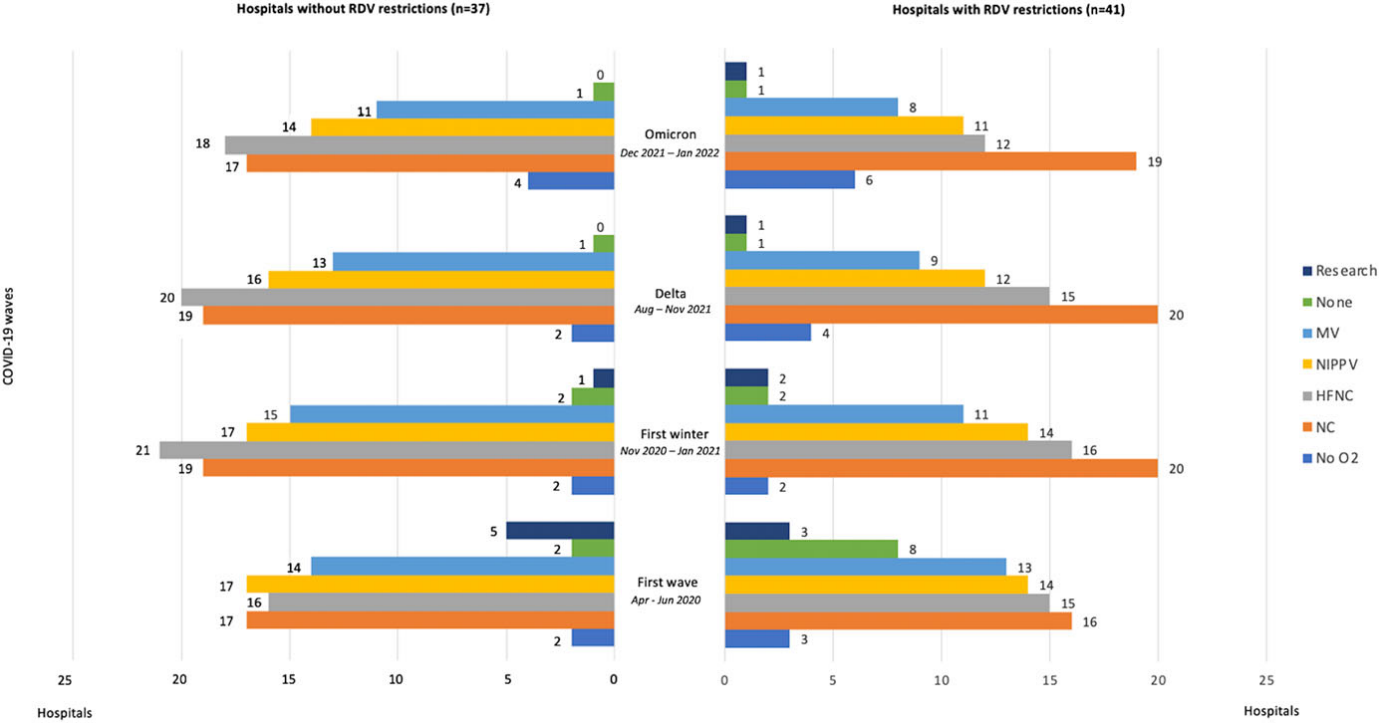
Remdesivir use by oxygen requirements in hospitals with (n = 41) and without (n = 37) remdesivir therapeutic restrictions.

In total, 41 respondents (53%) reported that RDV use was restricted by ASP. University hospitals (n = 19, 46%) were the most common type of hospitals with RDV restrictions and community hospitals (n = 19, 51%) were the most common in the group without restrictions. (Table 1). Among the 37 hospitals (47%) without RDV restrictions, RDV was reported to be commonly used for patients on MV, NIPPV, and HFNC (Fig. 1). RDV use was reported to be more commonly used for patients on HFNC than on LFNC during the first winter and the SARS-CoV-2 δ (delta) and ο (omicron) waves (Fig. 1). Overall, survey respondents from hospitals without restrictions reported less routine use of RDV for patients on MV with each surge. Survey respondents from the 41 hospitals (53%) with RDV restrictions reported routine RDV use for LFNC more commonly than for higher oxygen needs and used in LFNC more than hospitals without restrictions (Fig. 1). Use in MV, NIPPV and HFNC compared to LFNC decreased in the restricted group with each COVID-19 wave.

## Discussion

In this survey, we identified a wide gap between evidence-based guidelines and reported use of RDV. This gap was wider in hospitals without formulary restrictions for RDV. At hospitals without formulary restrictions, RDV was commonly allowed for MV despite robust evidence demonstrating a lack of benefit for these patients.^1–4,6^ Use in HFNC and NIPPV was also common. Importantly, we found that hospitals with formulary restrictions prioritized RDV use in patients with LFNC and did so more with each subsequent COVID-19 surge. Thus, formulary restrictions may have played an important role in guiding COVID-19 therapy during the pandemic. Physicians may have become more familiar with the guidelines and more comfortable with ASP recommendations as the pandemic progressed. Formulary restrictions can be enforced by pharmacy or stewardship and may also help educate clinicians when guidelines change. Restrictions were less common in community hospitals, which may reflect differences in staffing or expertise within ASP compared to university hospitals.

Implementation of novel therapeutics for COVID-19 has proved challenging for several reasons beyond adherence to guidelines. During the initial months of the pandemic, there was high demand for RDV and limited supply.^7^ Another key issue is cost. At a price of ∼$3,120 per RDV course,^8^ costs can be prohibitive to hospitals, particularly during COVID-19 surges, when COVID-19 case counts overwhelm hospitals, reimbursements for procedures diminishes, and the cost of personal protective equipment and nursing is significantly increased,^8,9^ Safety-net hospitals that serve communities of color and economically disenfranchised communities that have been disproportionately affected by COVID-19 are often particularly financially vulnerable.^10^ Unrestricted RDV use may lead to drug utilization in patients where there is no benefit at a cost that may be prohibitive for both patients and hospitals.

Our study had several limitations. Our convenience sample was small and may have been susceptible to bias because the survey was distributed through ID professional societies. In addition, many surveys were not completed for all COVID-19 waves. Nevertheless, our study provides valuable information on the use of RDV and the role of ASP.

Emerging infections present a continued challenge for clinicians. In this survey, we found a wide gap between evidence-based guidelines and RDV use. Formulary restrictions may help decrease this gap. ASP can play an important role in pandemic response.

## Supplementary material

For supplementary material accompanying this paper visit http://doi.org/10.1017/ash.2023.146. click here to view supplementary material
