# Supplementary material for: Remdesivir use and antimicrobial stewardship restrictions during the coronavirus disease 2019 (COVID-19) pandemic in the United States: A cross-sectional survey
Source: Antimicrob Steward Healthc Epidemiol. 2023 Mar 31;3(1):e63. doi: 10.1017/ash.2023.146 (PMC10127235; doi:10.1017/ash.2023.146)
Supplement: Supplementary file 1 [file ashsup.zip › S2732494X23001468sup002.pdf]

First wave

First wave: April, May, June 2020

Select the type of severity/patient in which each medication was used during the indicated period.

You can select several options for each medication if the drug is given to different levels of severity and oxygen needs.

Hydroxychloroquine
What kind of COVID-19 patients would routinely receive this medication?
* must provide value

- ☐ No oxygen needs
- ☐ Supplemental oxygen: Low flow nasal canula
- ☐ High flow nasal canula
- ☐ Non-invasive positive pressure ventilation (BIPAP)
- ☐ Mechanical ventilation
- ☐ NO USE during this period
- ☐ ONLY in the context of clinical trials

Convalescent plasma
What kind of COVID-19 patients would routinely receive this medication?
* must provide value

- ☐ No oxygen needs
- ☐ Supplemental oxygen: Low flow nasal canula
- ☐ High flow nasal canula
- ☐ Non-invasive positive pressure ventilation (BIPAP)
- ☐ Mechanical ventilation
- ☐ NO USE during this period
- ☐ ONLY in the context of clinical trials

Dexamethasone or other corticosteroids
What kind of COVID-19 patients would routinely receive this medication?
* must provide value

- ☐ No oxygen needs
- ☐ Supplemental oxygen: Low flow nasal canula
- ☐ High flow nasal canula
- ☐ Non-invasive positive pressure ventilation (BIPAP)
- ☐ Mechanical ventilation
- ☐ NO USE during this period
- ☐ ONLY in the context of clinical trials

Remdesivir
What kind of COVID-19 patients would routinely receive this medication?
* must provide value

- [ ] No oxygen needs
- [ ] Supplemental oxygen: Low flow nasal canula
- [ ] High flow nasal canula
- [ ] Non-invasive positive pressure ventilation (BIPAP)
- [ ] Mechanical ventilation
- [ ] NO USE during this period
- [ ] ONLY in the context of clinical trials

Tocilizumab or other IL-6 inhibitors
What kind of COVID-19 patients would routinely receive this medication?
* must provide value

- [ ] No oxygen needs
- [ ] Supplemental oxygen: Low flow nasal canula
- [ ] High flow nasal canula
- [ ] Non-invasive positive pressure ventilation (BIPAP)
- [ ] Mechanical ventilation
- [ ] NO USE during this period
- [ ] ONLY in the context of clinical trials

Baricitinib or other JAK inhibitors
What kind of COVID-19 patients would routinely receive this medication?
* must provide value

- [ ] No oxygen needs
- [ ] Supplemental oxygen: Low flow nasal canula
- [ ] High flow nasal canula
- [ ] Non-invasive positive pressure ventilation (BIPAP)
- [ ] Mechanical ventilation
- [ ] NO USE during this period
- [ ] ONLY in the context of clinical trials

First winter wave

First winter wave: *November, December 2020, and January 2021*

Select the type of severity/patient in which each medication was used during the indicated period.

You can select several options for each medication if the drug is given to different levels of severity and oxygen needs.

Hydroxychloroquine
What kind of COVID-19 patients would routinely receive this medication?
* must provide value

- ☐ No oxygen needs
- ☐ Supplemental oxygen: Low flow nasal canula
- ☐ High flow nasal canula
- ☐ Non-invasive positive pressure ventilation (BIPAP)
- ☐ Mechanical ventilation
- ☐ NO USE during this period
- ☐ ONLY in the context of clinical trials

Convalescent plasma
What kind of COVID-19 patients would routinely receive this medication?
* must provide value

- ☐ No oxygen needs
- ☐ Supplemental oxygen: Low flow nasal canula
- ☐ High flow nasal canula
- ☐ Non-invasive positive pressure ventilation (BIPAP)
- ☐ Mechanical ventilation
- ☐ NO USE during this period
- ☐ ONLY in the context of clinical trials

Dexamethasone or other corticosteroids
What kind of COVID-19 patients would routinely receive this medication?
* must provide value

- ☐ No oxygen needs
- ☐ Supplemental oxygen: Low flow nasal canula
- ☐ High flow nasal canula
- ☐ Non-invasive positive pressure ventilation (BIPAP)
- ☐ Mechanical ventilation
- ☐ NO USE during this period
- ☐ ONLY in the context of clinical trials

Remdesivir
What kind of COVID-19 patients would routinely receive this medication?
* must provide value

- ☐ No oxygen needs
- ☐ Supplemental oxygen: Low flow nasal canula
- ☐ High flow nasal canula
- ☐ Non-invasive positive pressure ventilation (BIPAP)
- ☐ Mechanical ventilation
- ☐ NO USE during this period
- ☐ ONLY in the context of clinical trials

Tocilizumab or other IL-6 inhibitors
What kind of COVID-19 patients would routinely receive this medication?
* must provide value

- ☐ No oxygen needs
- ☐ Supplemental oxygen: Low flow nasal canula
- ☐ High flow nasal canula
- ☐ Non-invasive positive pressure ventilation (BIPAP)
- ☐ Mechanical ventilation
- ☐ NO USE during this period
- ☐ ONLY in the context of clinical trials

Baricitinib or other JAK inhibitors
What kind of COVID-19 patients would routinely receive this medication?
* must provide value

- ☐ No oxygen needs
- ☐ Supplemental oxygen: Low flow nasal canula
- ☐ High flow nasal canula
- ☐ Non-invasive positive pressure ventilation (BIPAP)
- ☐ Mechanical ventilation
- ☐ NO USE during this period
- ☐ ONLY in the context of clinical trials

Delta **wave**

Delta wave: *August, September, October, November 2021*

Select the type of severity/patient in which each medication was used during the indicated period.

You can select several options for each medication if the drug is given to different levels of severity and oxygen needs.

Hydroxychloroquine
What kind of COVID-19 patients would routinely receive this medication?
* must provide value

- ☐ No oxygen needs
- ☐ Supplemental oxygen: Low flow nasal canula
- ☐ High flow nasal canula
- ☐ Non-invasive positive pressure ventilation (BIPAP)
- ☐ Mechanical ventilation
- ☐ NO USE during this period
- ☐ ONLY in the context of clinical trials

Convalescent plasma
What kind of COVID-19 patients would routinely receive this medication?
* must provide value

- ☐ No oxygen needs
- ☐ Supplemental oxygen: Low flow nasal canula
- ☐ High flow nasal canula
- ☐ Non-invasive positive pressure ventilation (BIPAP)
- ☐ Mechanical ventilation
- ☐ NO USE during this period
- ☐ ONLY in the context of clinical trials

Dexamethasone or other corticosteroids
What kind of COVID-19 patients would routinely receive this medication?
* must provide value

- ☐ No oxygen needs
- ☐ Supplemental oxygen: Low flow nasal canula
- ☐ High flow nasal canula
- ☐ Non-invasive positive pressure ventilation (BIPAP)
- ☐ Mechanical ventilation
- ☐ NO USE during this period
- ☐ ONLY in the context of clinical trials

Remdesivir
What kind of COVID-19 patients would routinely receive this medication?
* must provide value

- ☐ No oxygen needs
- ☐ Supplemental oxygen: Low flow nasal canula
- ☐ High flow nasal canula
- ☐ Non-invasive positive pressure ventilation (BIPAP)
- ☐ Mechanical ventilation
- ☐ NO USE during this period
- ☐ ONLY in the context of clinical trials

Tocilizumab or other IL-6 inhibitors
What kind of COVID-19 patients would routinely receive this medication?
* must provide value

- ☐ No oxygen needs
- ☐ Supplemental oxygen: Low flow nasal canula
- ☐ High flow nasal canula
- ☐ Non-invasive positive pressure ventilation (BIPAP)
- ☐ Mechanical ventilation
- ☐ NO USE during this period
- ☐ ONLY in the context of clinical trials

Baricitinib or other JAK inhibitors
What kind of COVID-19 patients would routinely receive this medication?
* must provide value

- ☐ No oxygen needs
- ☐ Supplemental oxygen: Low flow nasal canula
- ☐ High flow nasal canula
- ☐ Non-invasive positive pressure ventilation (BIPAP)
- ☐ Mechanical ventilation
- ☐ NO USE during this period
- ☐ ONLY in the context of clinical trials

Omicron **wave**

Omicron **wave:** *December 2021, January 2022*

Select the type of severity/patient in which each medication was used during the indicated period.

You can select several options for each medication if the drug is given to different levels of severity and oxygen needs.

Hydroxychloroquine
What kind of COVID-19 patients would routinely receive this medication?
* must provide value

- ☐ No oxygen needs
- ☐ Supplemental oxygen: Low flow nasal canula
- ☐ High flow nasal canula
- ☐ Non-invasive positive pressure ventilation (BIPAP)
- ☐ Mechanical ventilation
- ☐ NO USE during this period
- ☐ ONLY in the context of clinical trials

Convalescent plasma
What kind of COVID-19 patients would routinely receive this medication?
* must provide value

- ☐ No oxygen needs
- ☐ Supplemental oxygen: Low flow nasal canula
- ☐ High flow nasal canula
- ☐ Non-invasive positive pressure ventilation (BIPAP)
- ☐ Mechanical ventilation
- ☐ NO USE during this period
- ☐ ONLY in the context of clinical trials

Dexamethasone or other corticosteroids
What kind of COVID-19 patients would routinely receive this medication?
* must provide value

- ☐ No oxygen needs
- ☐ Supplemental oxygen: Low flow nasal canula
- ☐ High flow nasal canula
- ☐ Non-invasive positive pressure ventilation (BIPAP)
- ☐ Mechanical ventilation
- ☐ NO USE during this period
- ☐ ONLY in the context of clinical trials

Remdesivir
What kind of COVID-19 patients would routinely receive this medication?
* must provide value

- ☐ No oxygen needs
- ☐ Supplemental oxygen: Low flow nasal canula
- ☐ High flow nasal canula
- ☐ Non-invasive positive pressure ventilation (BIPAP)
- ☐ Mechanical ventilation
- ☐ NO USE during this period
- ☐ ONLY in the context of clinical trials

Tocilizumab or other IL-6 inhibitors
What kind of COVID-19 patients would routinely receive this medication?
* must provide value

- ☐ No oxygen needs
- ☐ Supplemental oxygen: Low flow nasal canula
- ☐ High flow nasal canula
- ☐ Non-invasive positive pressure ventilation (BIPAP)
- ☐ Mechanical ventilation
- ☐ NO USE during this period
- ☐ ONLY in the context of clinical trials

Baricitinib or other JAK inhibitors
What kind of COVID-19 patients would routinely receive this medication?
* must provide value

- ☐ No oxygen needs
- ☐ Supplemental oxygen: Low flow nasal canula
- ☐ High flow nasal canula
- ☐ Non-invasive positive pressure ventilation (BIPAP)
- ☐ Mechanical ventilation
- ☐ NO USE during this period
- ☐ ONLY in the context of clinical trials
