# Supplementary material for: Remdesivir use and antimicrobial stewardship restrictions during the coronavirus disease 2019 (COVID-19) pandemic in the United States: A cross-sectional survey
Source: Antimicrob Steward Healthc Epidemiol. 2023 Mar 31;3(1):e63. doi: 10.1017/ash.2023.146 (PMC10127235; doi:10.1017/ash.2023.146)
Supplement: Supplementary file 1 [file ashsup.zip › S2732494X23001468sup003.pdf]

## COVID-19 Management Survey

**Survey of COVID19 management at different stages**

Throughout the pandemic, the management of COVID-19 has evolved. Hospitals around the world have adapted to rapidly evolving literature. Help us define the management of COVID-19 in different phases and waves of the pandemic with this short survey.

This survey is anonymous.

**What city is your hospital located?** ____________________

**How many beds does your hospital have?**
* must provide value

- ○ < 200
- ○ 201-300
- ○ 301-400
- ○ >400

**What best describes your hospital?**
* must provide value

- ○ Community hospital
- ○ County/public hospital
- ○ University-affiliated Community-Based Teaching Hospital
- ○ University-based Teaching Hospital
- ○ Veterans Administration Medical Center
- ○ Other

**If other, please type here:** ____________________

**What is your role?**
* must provide value

- ○ Physician (Hospitalist, Internist, Family Medicine)
- ○ Infectious Diseases Physician/Consultant
- ○ Pharmacist
- ○ Other

**If other, please type here:**

**Does your hospital have an active antimicrobial stewardship program?**
* must provide value

- ◯ Yes
- ◯ No

**Has your hospital restricted the use of any COVID-19 therapies?**
* must provide value

- ☐ Convalescent plasma
- ☐ Remdesivir
- ☐ Baricitinib or other JAK-2 inhibitors
- ☐ Tocilizumab or other IL-6 inhibitors
- ☐ None

**If your hospital has restricted therapies, who in your hospital is able to order or approve restricted COVID-19 therapies?**

- ☐ Pharmacist
- ☐ Infectious diseases (approve, order, or via consultation)
- ☐ Pulmonary/Critical care (approve, order, or via consultation)
- ☐ Designated hospitalist (approve, order, or via consultation)
- ☐ Designated COVID-19 team (hospitalist, unit or group of physicians)
- ☐ Other

**If other, please type here:**
