# Supplementary figures and images for: Remdesivir use and antimicrobial stewardship restrictions during the coronavirus disease 2019 (COVID-19) pandemic in the United States: A cross-sectional survey
Source: Antimicrob Steward Healthc Epidemiol. 2023 Mar 31;3(1):e63. doi: 10.1017/ash.2023.146 (PMC10127235; doi:10.1017/ash.2023.146)

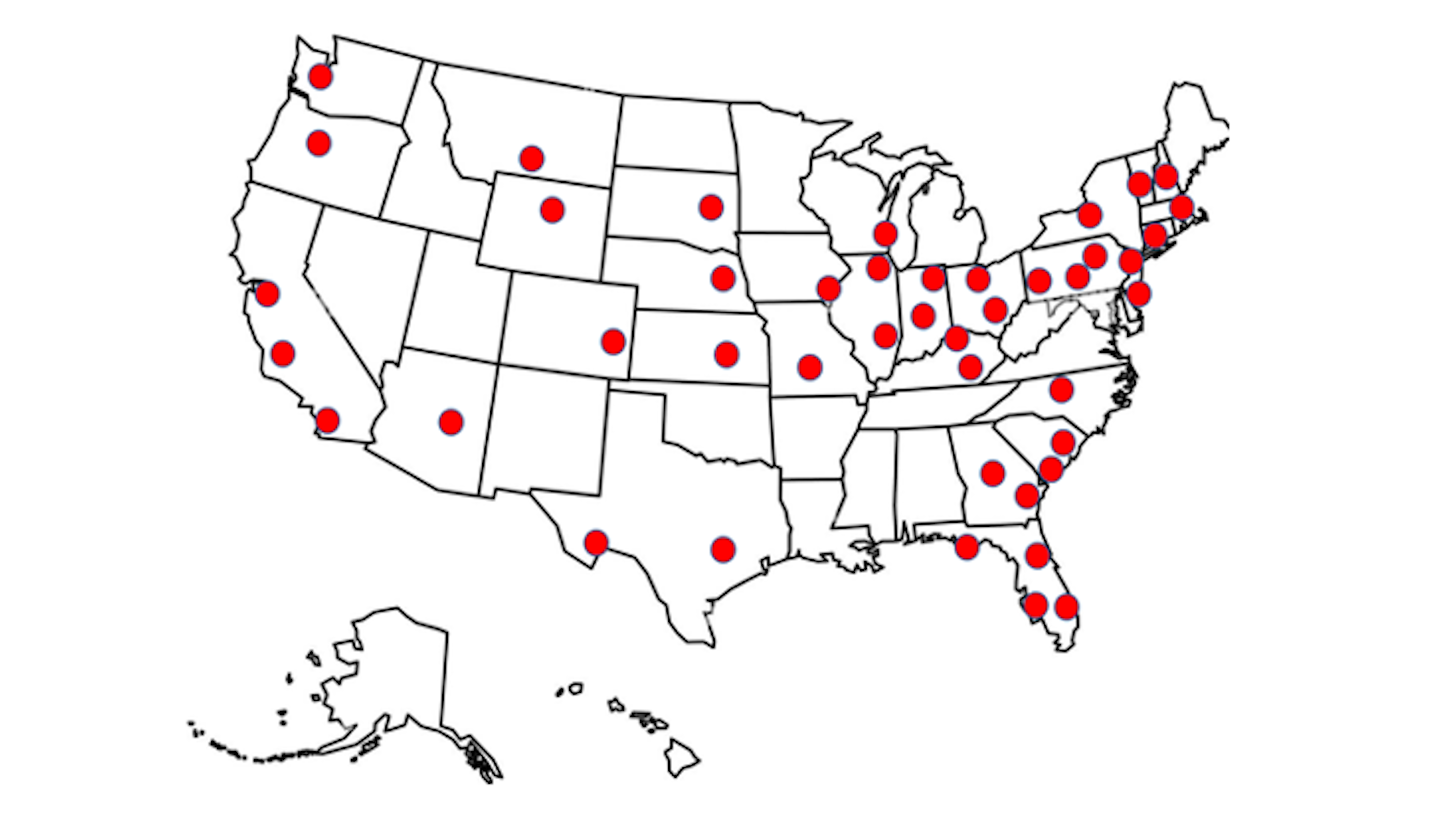

Supplement: Supplementary file 1 [file ashsup.zip › S2732494X23001468sup001.tif]
